# Is quantitative pupillometry affected by ambient light? A prospective crossover study

**DOI:** 10.1007/s10877-025-01293-z

**Published:** 2025-04-10

**Authors:** Sanna Holmskär, Malin Öhrn, Moa Furudahl, Johannes Kesti, Jakob Pansell

**Affiliations:** 1 The Department of Anesthesia and Intensive Care Medicine, Karolinska University Hospital, Stockholm, Sweden; 2 The Department of Clinical Neuroscience, Karolinska Institutet, Stockholm, Sweden

**Keywords:** Quantitative Pupillometry, Brain Injuries, Neurology, Neurosurgery, Critical Care, Stroke, Cardiac Arrest, Brain Edema, Cerebral Edema, Intensive Care

## Abstract

Purpose: Pupillary examination is a central part of the neurological assessment. While quantitative pupillometry (QP) improves reliability, the impact of ambient light, particularly on the Neurological Pupil index (NPi), remains unclear. This study aimed to clarify the effects of ambient light on QP parameters in a critical care setting. Methods: We performed a prospective crossover study, including 20 adult patients requiring invasive ventilation. Pupillometry was performed during bright condition (BC1), then dark condition (DC), then bright condition again (BC2). In our primary analysis we compared NPi values across conditions (DC1 vs. BC, BC vs. DC2, DC1 vs. DC2). In the secondary analysis, we compared all other QP parameters. Results: All QP values except constriction velocity and dilation velocity were non-normal. The median NPi was significantly lower in BC compared to dark conditions DC1 in both eyes. In 25% of participants the NPi decreased by 0.6 or more. Conversely, a significant increase in median NPi of both eyes was observed when switching from bright conditions back to dark (BC vs. DC2). No significant difference was found between the two dark condition measurements (DC1 and DC2). The secondary analysis showed that the differences in NPi were driven by differences in most, but not all, QP parameters included in NPi. Conclusions: We corroborate previous findings that the level of ambient light affects QP parameters in critically ill patients. This needs to be considered for accurate interpretation of QP parameters. Future studies may explore potential automated light correction methods for wider clinical applicability.

## Introduction

Examination of the pupillary size and reflexes are fundamental elements of the neurological exam. Traditionally, examination of pupil size and the pupillary light reflex (PLR) have been performed manually with a penlight. This technique is affected by substantial interrater variability, shows low consistency and is described in subjective and descriptive terms [1]. Quantitative pupillometry (QP) has been developed to address these issues and provide quantitative values of PLR. It is an automated technique that is highly reproducible, easy to use and shows excellent interrater reliability [2, 3]. In QP, the pupil is video recorded before, during and after illumination with visible light [4]. Quantitative pupillometry presents numeric values for pupillary size, relative pupillary constriction in percentage, latency and velocity of PLR and of pupillary dilation after PLR. The Neuroptics^®^ NPi-200 (Neuroptics, Irvine CA, USA) pupillometer calculates a composite value of all these values, called the Neurological Pupil index (NPi). The NPi ranges from 0 to 5 with ≥ 3 as a threshold for normal pupillary responses. The NPi is reportedly more robust than manual PLR examination to confounding by factors such as pharmacological effects [2]. Manual PLR examination is largely affected by ambient light conditions, with the pupils being naturally smaller in brighter conditions and the disagreement between raters using manual PLR examination increasing in smaller pupils [3]. The effects of ambient light on the NPi remain largely unknown. There is only one previous study, including seven healthy volunteers and seven critical care patients, that evaluates the NPi as measured by the Neuroptics^®^ NPi-200 (Neuroptics, Irvine CA, USA) pupillometer under different ambient light conditions. That study showed a significant effect of ambient light on all NPi values except latency, but including NPi, in the critical care patients [5]. Due to the increasing use of the NPi as part of the neurological exam in large patient groups, the effects of ambient light need to be further elucidated. This study aims to do that.

## Methods

This prospective study was performed in the neurointensive care unit and the general intensive care unit at the Karolinska University Hospital in Stockholm, Sweden. We performed a sample size calculation based on the effect size shown by Ong et al. [5]. This yielded a necessary sample size of 10 patients to demonstrate a similar NPi difference, with a power of 80% and the level of statistical significance set at *p* < 0.05. To account for uncertainty due to a very small sample size in that study, we decided to include 20 patients. Inclusion criteria were age ≥ 18 years, unconscious or sedated, and treated with invasive ventilation in the intensive care unit. Exclusion criteria were ocular trauma or disease affecting the pupillary reaction, or ICP fluctuations exceeding ± 2 mmHg during the measurement session, in patients with invasive ICP monitoring. We used a convenience sample, including all eligible patients that were available for a measurement session without delaying or affecting planned care or interventions, when one of the nurses gathering data were available to perform measurements.

Pupillometry under dark conditions (DC1) was performed with all electrical lights switched off, the shades closed and the door closed. To avoid differences in outdoor light leaking through the shades, all data was gathered after dark. Pupillometry under bright conditions (BC) were then performed with the overhead lights in the ceiling turned full on, as during normal nursing interventions. To complete the crossover design, the lights were then switched off and pupillometry was performed again, during dark conditions (DC2). These standardized lighting conditions achieve an illuminance of 1 lx under DC and 301 lx under BC, measured post-hoc during mimicked study conditions, at the head of the bed with a Hagner^®^ Universal Photometer/Radiometer Model S4. Pupillometry bservations were performed with intervals of two minutes. The patients’ eyelids were closed before and between observations, and the lights were switched off for five minutes prior to the first observation. In the case of pharmacological interventions or other stimuli occurring between observations, the observation series was aborted. Pupillometry was performed twice each at DC1, BC and DC2. As recommended by Kelbsch et al. [6], we excluded the first observation in the series, by performing an extra round of observations at DC1.

Pupillometry was performed with the The Neuroptics^®^ NPi-200 (Neuroptics, Irvine CA, USA) and gathered by two of the authors in January and February 2024. We gathered all output data from the pupillometer: NPi, maximum and minimum pupil size (Size and Min) in millimeters, relative change in pupil size (CH) in percents, constriction velocity (CV) in mm/s, maximum constriction velocity (MCV) in mm/s, latency (LAT) in seconds and dilation velocity (DV) in mm/s. Descriptive data for the cohort was gathered from electronic patient charts.

### Statistical analysis

All data was tested for normality with the Shapiro Wilks test. Continuous data were reported as means with standard deviations or as medians with interquartile ranges, as proper with regards to the distributions. Binary data were reported as frequencies. In the primary analysis, we used the first set of observations at DC1, BC and DC2. We compared NPi in pairs of DC1 and BC, BC and DC2, and DC1 and DC2. Differences in NPi were tested for significance with either paired t-tests or paired Wilcoxon signed-rank tests, depending on the distributions. We hypothesized that an effect of ambient light on the NPi should result in significant differences between DC1 and BC as well as between BC and DC2, but no difference between DC1 and DC2. The level of statistical significance was set at a *p*-value < 0.05.

In the secondary analysis, we performed similar analyses to explore differences in all other documented pupillometry values between DC1 and BC, BC and DC2, and DC1 and DC2. We also analyzed intra-individual variability separately at DC1, BC and DC2. This was performed by paired comparisons of the repeated observations of NPi within DC1, BC and DC2 respectively. These within individual differences were then compared to the differences in NPi between lighting conditions, to explore if random differences in NPi regardless of differences in ambient light could explain potential differences in NPi between DC1, BC and DC2. Finally, we performed a sensitivity analysis, removing subjects with an abnormal NPi at baseline and re-running the primary analysis.

### Ethical considerations

The study was conducted in accordance with the Helsinki declaration and was approved by the Swedish Ethical Review Authority, record number 2022-03045-01. The requirement for informed consent from the study subjects was waived by the Swedish Ethical Review Authority due to the nature of the cohort that makes informed consent unfeasible. We informed the patients´ next of kin and gave them right to opt out on behalf of the patient.

## Results

We included 20 patients of which 12 were female. Half of the patients were previously healthy. The most common comorbidity was cardiovascular disease (50%) and the most common intensive care diagnoses were traumatic brain injury (30%) and subarachnoid hemorrhage (25%). See Table 1 for baseline characteristics.

**Table 1:** Descriptive data for the cohort

Female *n*/*N* (%)	12/20 (60)
Age, median (interquartile range)	57 (38, 68)
**Comorbidities**	
None, n/N (%)	10/20 (50)
Cardiovascular disease, n/N (%)	10/20 (50)
Diabetes, n/N (%)	2/20 (10)
Ischemic stroke, n/N (%)	1/20 (5)
Other neurological disease, n/N (%)	1/20 (5)
**Intensive care diagnosis**	
Traumatic brain injury, n/N (%)	6/20 (30)
Subarachnoid hemorrhage, n/N (%)	5/20 (25)
Cardiac arrest, n/N (%)	3/20 (15)
Ischemic stroke, n/N (%)	3/20 (15)
Respiratory failure, n/N (%)	2/20 (10)
Postoperative hemorrhage, n/N (%)	1/20 (5)
**Treatment**	
Propofol infusion	17/20 (85)
Midazolam infusion	7/20 (35)
Opioid infusion	17/20 (85)
Vasopressor infusion	17/20 (85)
Invasive monitoring of intracranial pressure	11/20 (55)

Participation was not withdrawn for any of the subjects. No measurement sessions were aborted due to ICP fluctuations, pharmacological interventions or other stimuli.

Constriction velocity and dilation velocity were the only QP data that were normally distributed. They were compared between levels of ambient light by paired t-tests. All other QP parameters were non-normal and reported as medians with interquartile ranges and compared by paired Wilcoxon signed-rank tests. Baseline NPi was < 3 bilaterally in one patient and in the left eye in one patient. The median NPi in DC1 was 4.6 on the right side (IQR 4.5; 4.7) and 4.7 on the left side (IQR 4.4; 4.7). In DC2, the median NPi was 4.5 on the right side (IQR 4.4; 4.7) and 4.6 on the left side (IQR 4.4; 4.7). There was no significant difference between DC1 and DC2 (right side *p* = 0.89, left side *p* = 0.16).

**Fig. 1:**
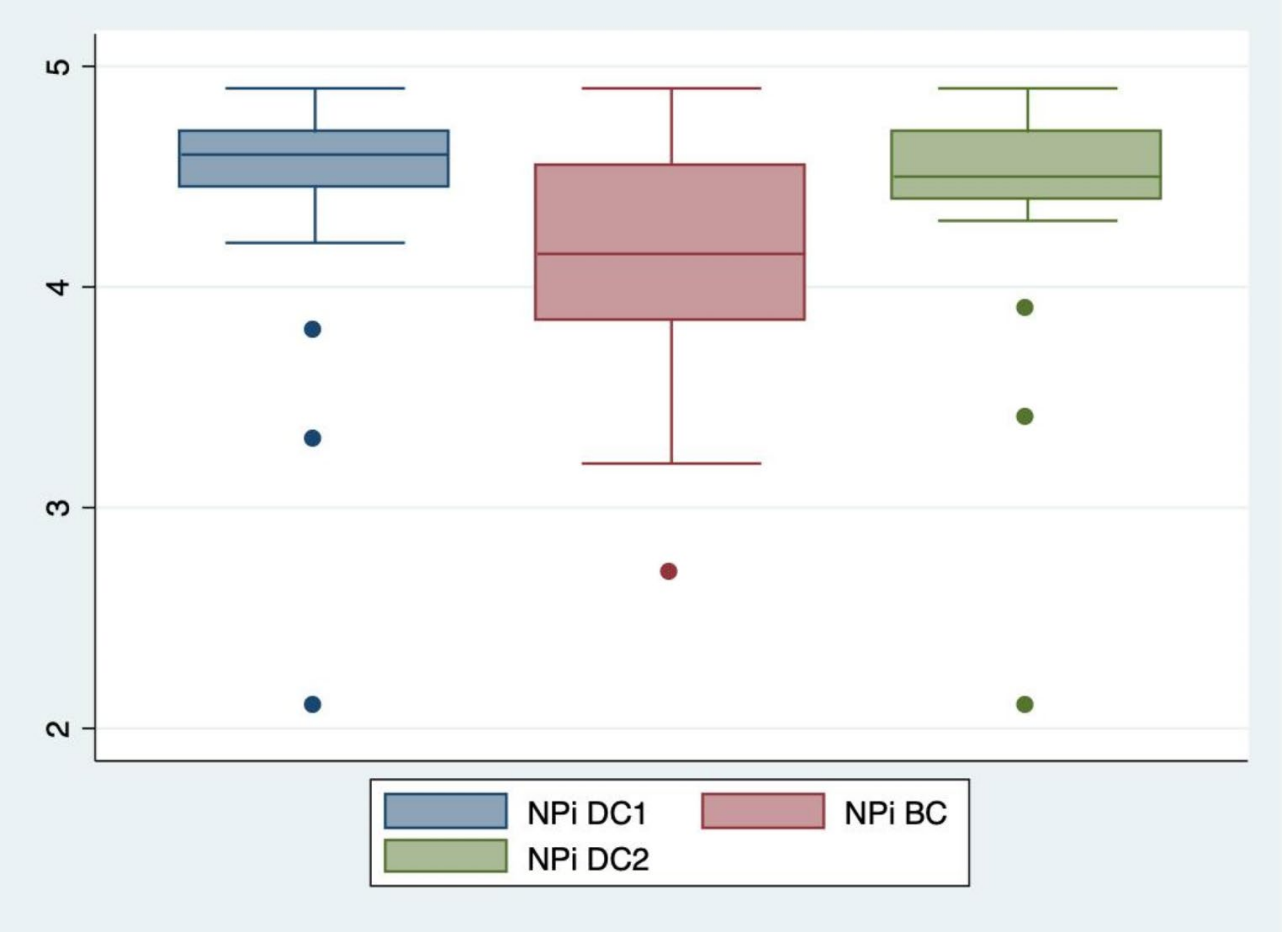
Right eye NPi The Neurological Pupil index (NPi) of the right eyes during dark conditions (DC1), bright conditions (BC) and then dark conditions again (DC2). One measurement each in 20 patients

**Fig. 2:**
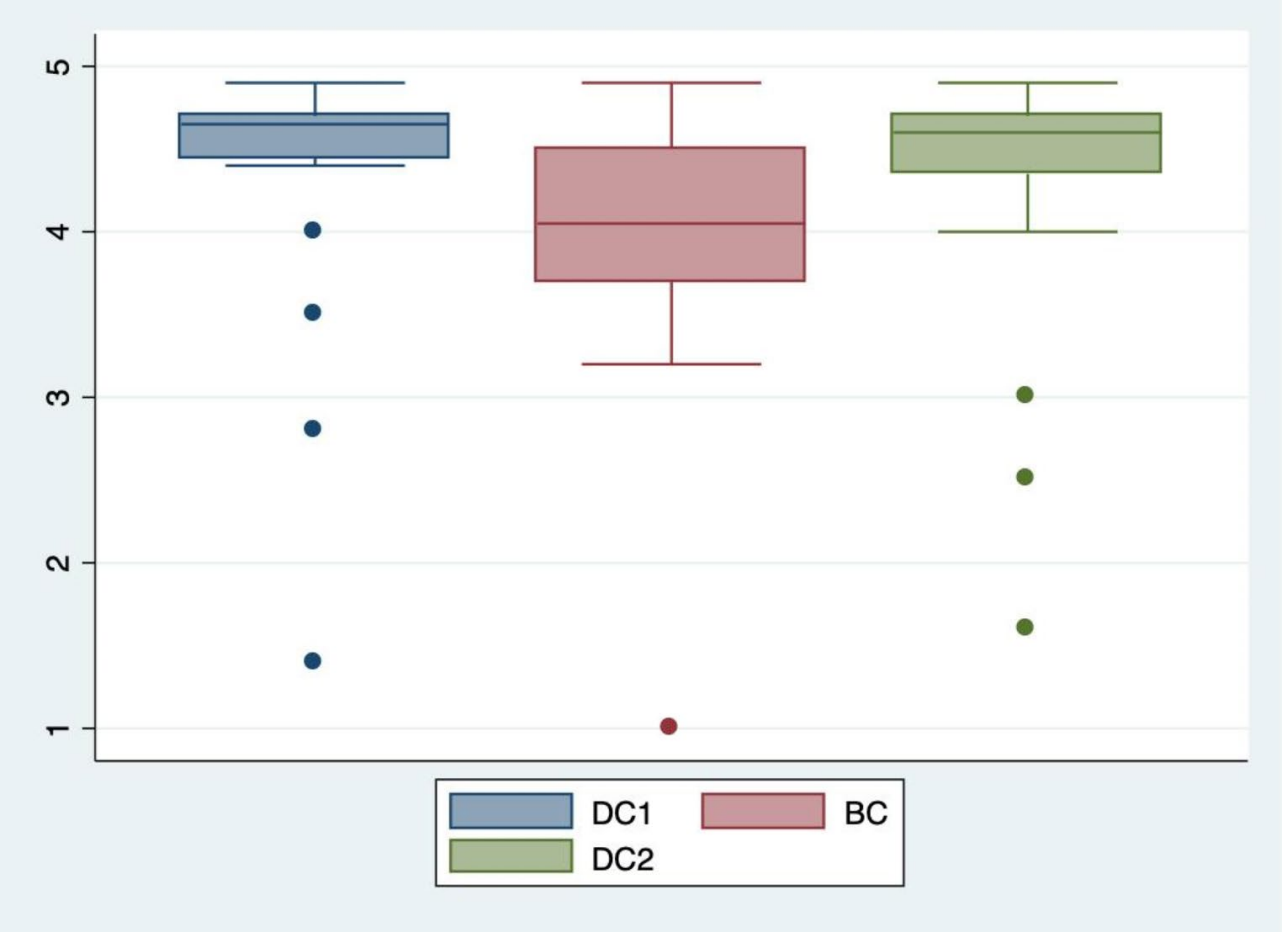
Left eye NPi The Neurological Pupil index (NPi) of the left eyes during dark conditions (DC1), bright conditions (BC) and then dark conditions again (DC2). One measurement each in 20 patients

**Fig. 3:**
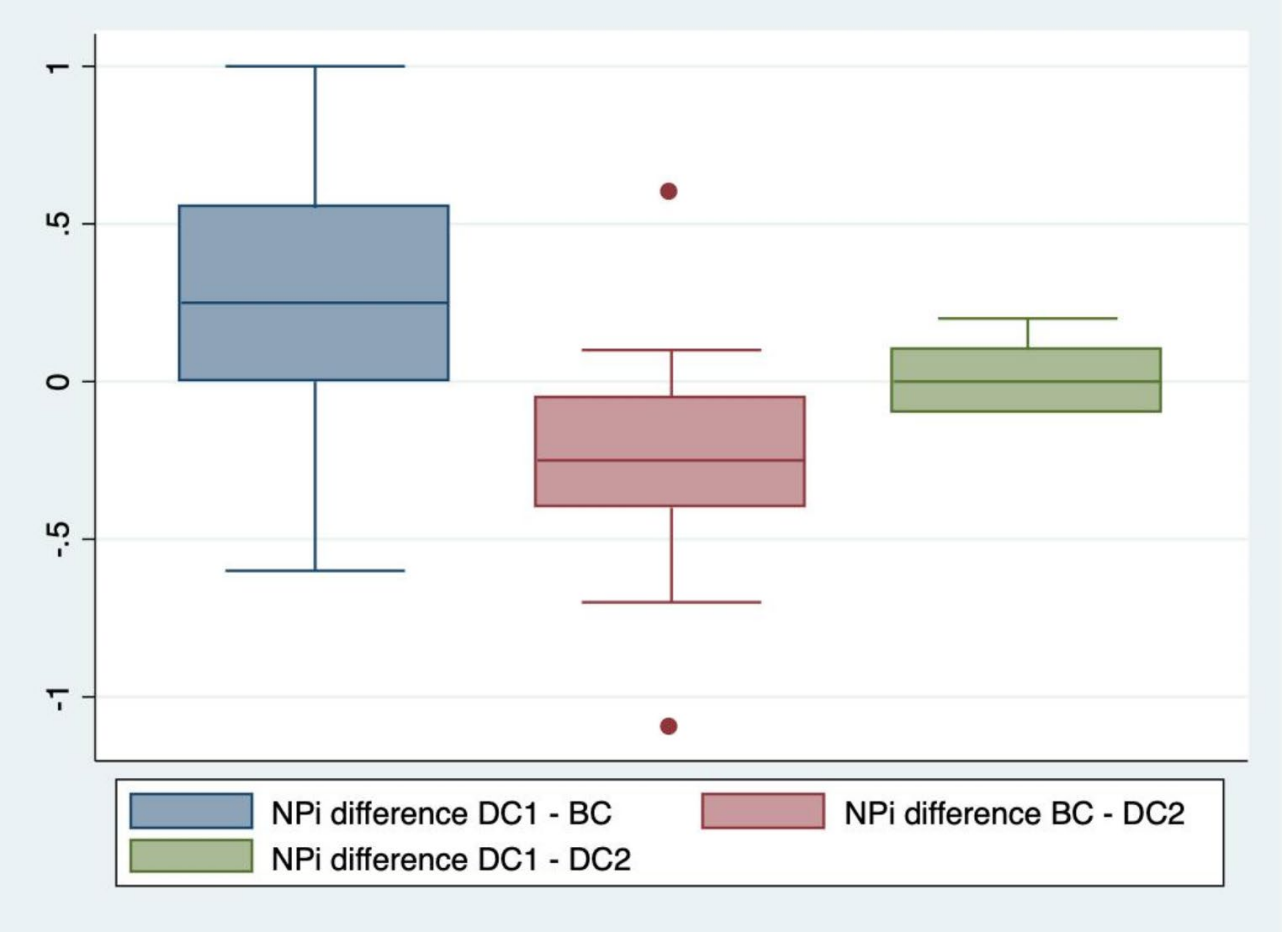
Changes in NPi in the right eyes The difference in Neurological Pupil index (NPi) in the right eyes between the first session of dark conditions (DC1) and bright conditions (BC), between BC and the second session of dark conditions (DC2), and finally between DC1 and DC2. Based on one measurement each in 20 patients per DC1, BC and DC2

**Fig. 4:**
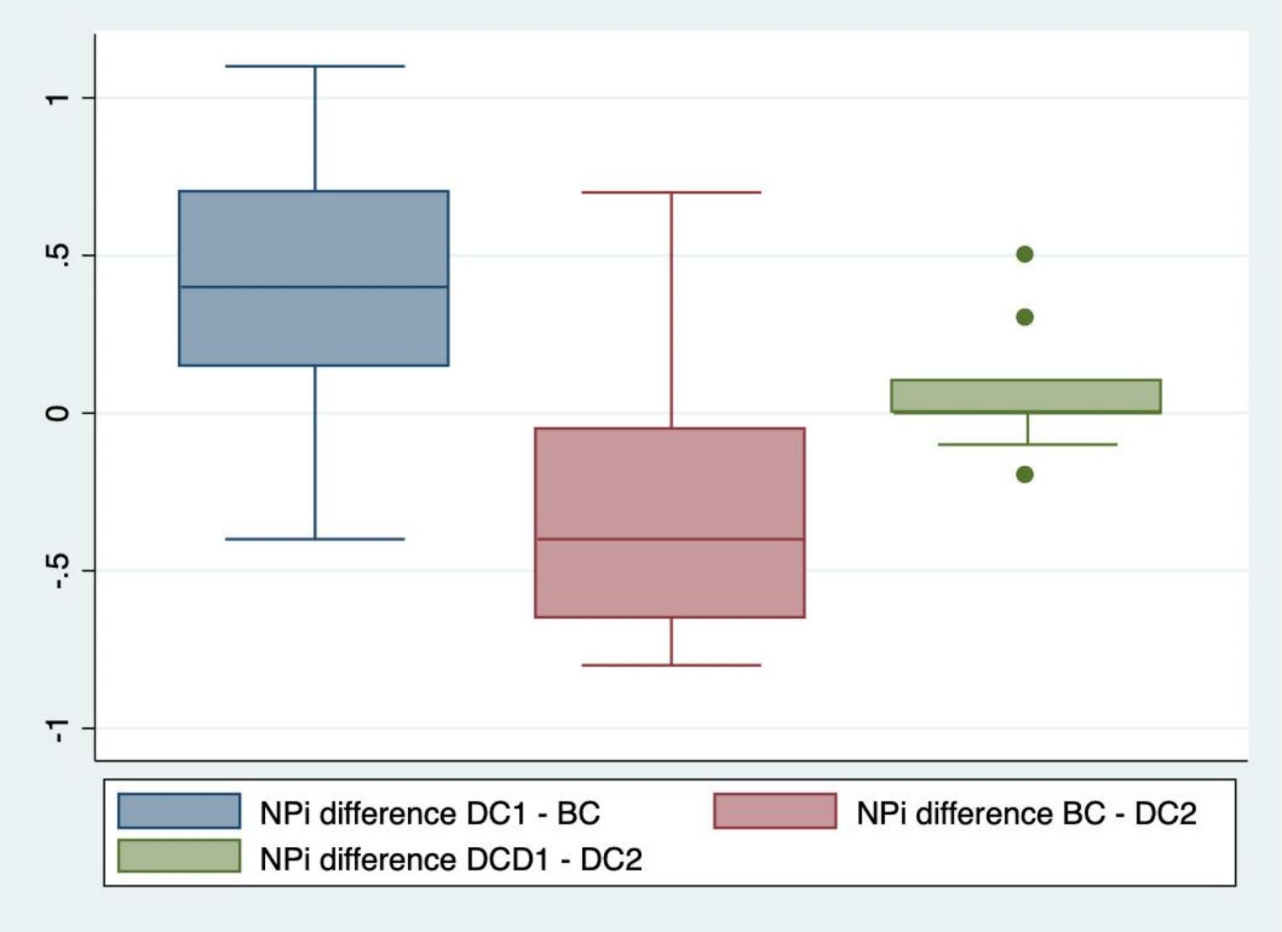
Changes in NPi in the left eyes The difference in Neurological Pupil index (NPi) in the left eyes between the first session of dark conditions (DC1) and bright conditions (BC), between BC and the second session of dark conditions (DC2), and finally between DC1 and DC2. Based on one measurement each in 20 patients per DC1, BC and DC2

The median NPi in BC was 4.2 on the right side (IQR 3.9; 4.6). The median difference in NPi for the right eye was a significant 0.3 (IQR 0.0; 0.6) decrease between DC1 and BC (*p* = 0.01). The right side NPi decreased in 15 out of 20 subjects (75%) between DC1 and BC. In five patients (25%) the right side NPi decreased by 0.6 or more. On the left side, the median NPi in BC was 4.1 (IQR 3.7; 4.5). The median difference in NPi for the left side was a significant 0.4 (IQR 0.2; 0.7) decrease between DC1 and BC (*p* < 0.001). The left side NPi decreased in 17 out of 20 subjects (85%). In five patients (25%) the left side NPi decreased by 0.7 or more. The median difference between BC and DC2 was a significant 0.3 increase (IQR 0.1; 0.4) on the right side (*p* < 0.001) and a significant 0.4 (IQR 0.1; 0.7) increase on the left side (*p* < 0.001). See Figs. 1, 2, 3 and 4.

**Fig. 5:**
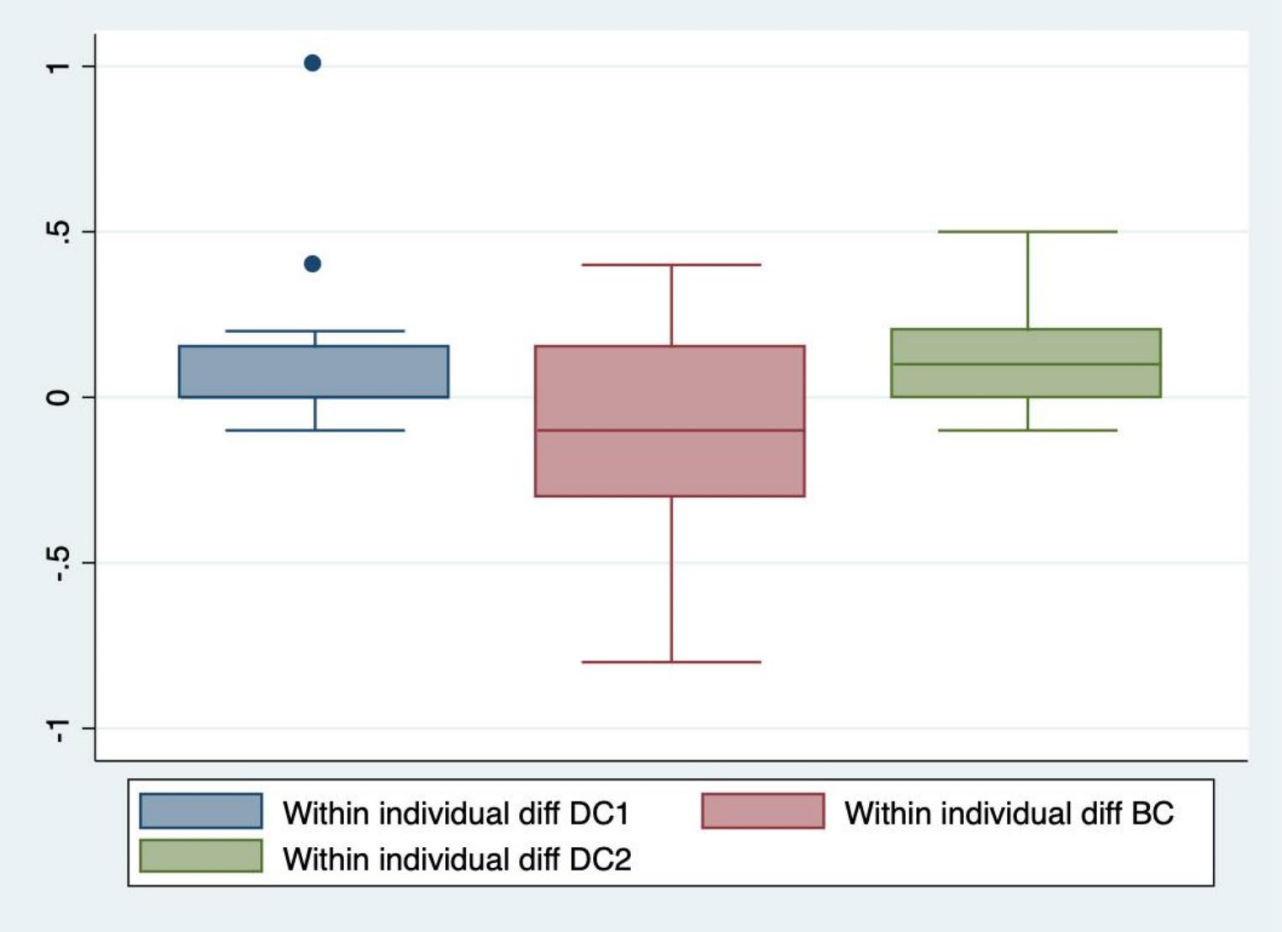
Within individual differences in NPi at DC1, BC and DC2 in right eyes The difference (diff) in Neurological Pupil index (NPi) in the right eyes within the first session of dark conditions (DC1), within bright conditions (BC), and within the second session of dark conditions (DC2)

**Fig. 6:**
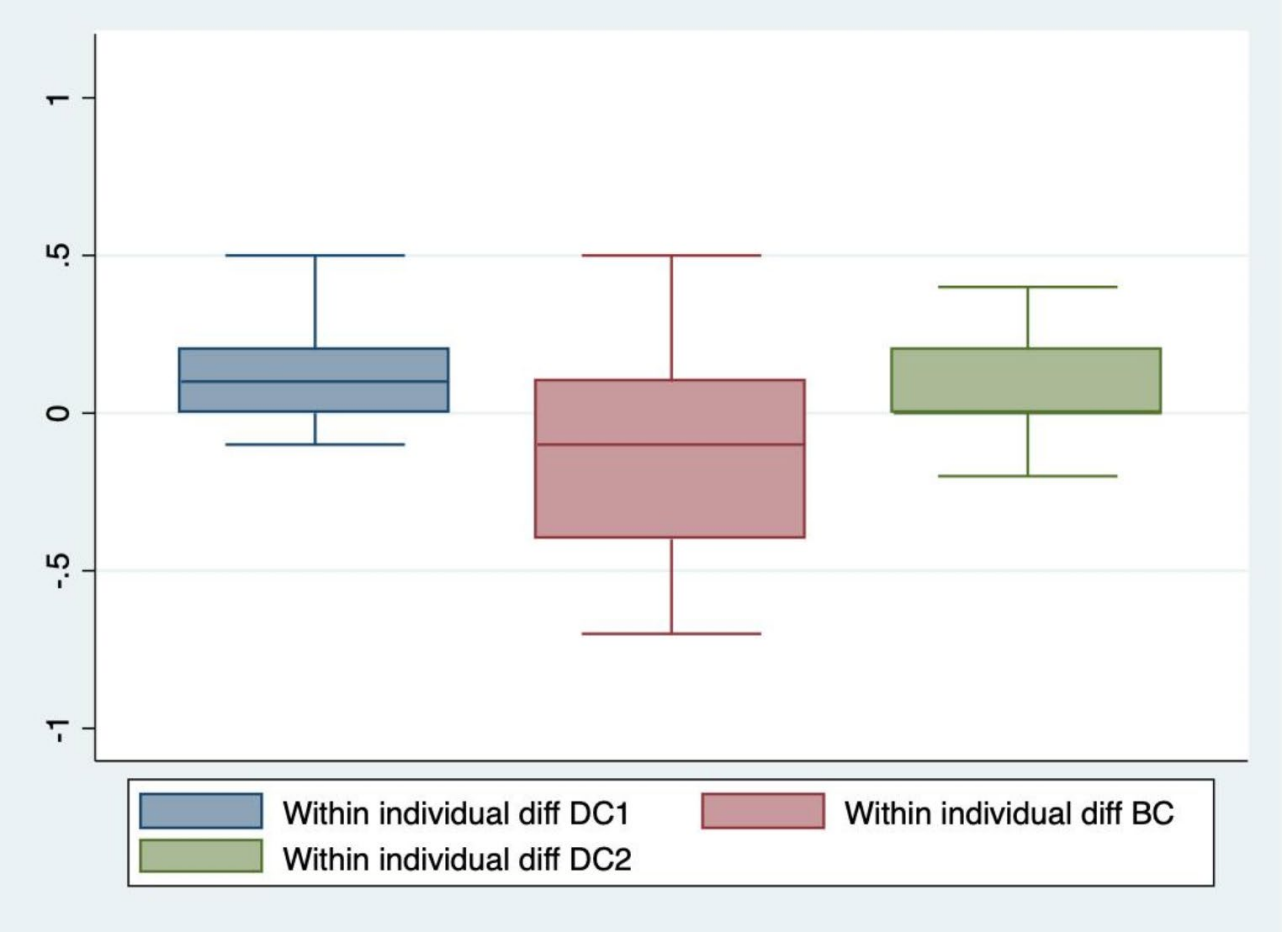
Within individual differences in NPi at DC1, BC and DC2 in left eyes The difference (diff) in Neurological Pupil index (NPi) in the left eyes within the first session of dark conditions (DC1), within bright conditions (BC), and within the second session of dark conditions (DC2)

The secondary analysis showed significant differences between dark and bright conditions for all parameters except latency and minimum pupil size bilaterally, and dilation velocity on the right side. These parameters did not differ significantly between any of the light conditions. There were no significant differences in any of the parameters between DC1 and DC2. See Table 2. The within individual differences in NPi at DC1, BC and DC2 respectively are shown in Figs. 5 and 6. The median intra-individual differences in NPi at DC1 were 0 (IQR 0.0; 0.2) for both eyes. The median intra-individual differences in NPi at BC were 0.1 (IQR − 0.3; 0.2) for the right eye and − 0.1 (IQR − 0.4; 0.1) for the left eye. The median intra-individual differences in DC2 were 0.1 (IQR 0; 0.2) for the right eye and 0 (IQR 0; 0.2) for the left eye. All within individual differences in NPi at DC1, BC and DC2 were significantly smaller than the differences DC1-BC and BC-DC2. Means or medians for pupillometry values at each observation are reported in Table 3. In the two patients with abnormal NPi in at least one eye at baseline, changes in pupillometry values were similar, though the small number does not allow for any statistical analysis of this. The sensitivity analysis, excluding these subjects from the primary analysis, did not change overall results of the differences in pupillometry values between lighting conditions. In the sensitivity analysis, the median decrease in NPi between DC1 and BC for the right and left eyes respectively were 0.3 (IQR 0.0; 0.5) and 0.4 (0.2; 0.7), the median increase between BC and DC2 for the right and left eyes respectively were 0.3 (0.4; 0.1) and 0.4 (0.7; 0.1), and the median difference between DC1 and DC2 for the right and left eyes respectively were 0.0 (-0.1; 0.1) and 0.1 (0.0; 0.1).

**Table 2:** Secondary analysis of differences in pupillometry values. Note that differences are reported as means or medians of the differences, not as differences of the means or medians. DC1.1 first observation under first set of dark conditions, BC1.1 first observation under bright conditions, DC2.1 first observation under second set of dark conditions, IQR interquartile range, NPi neurological pupil index, SD standard deviation

Parameter	DC1.1	BC1.1	DC2.1	Difference DC1-BC	Difference BC-DC2	Difference DC1- DC2
Right side NPi, median (IQR)	4.6 (4.5, 4.7)	4.2 (3.9, 4.6)	4.5 (4.4, 4.7)	0.3 (0.0, 0.6)	-0.3 (-0.4, -0.1)	0.0 (-0.1, 0.1)
Left side NPi, median (IQR)	4.7 (4.4, 4.7)	4.1 (3.7, 4.5)	4.6 (4.4, 4.7)	0.4 (0.2, 0.7)	-0.4 (-0.7, -0.1)	0.0 (0.0, 0.1)
Right side Maximum pupil size, mm, median (IQR)	2.42 (2.23, 3.03)	2.29 (2.02, 2.79)	2.41 (2.17, 3.16)	0.17 (0.09, 0.47) *p* < 0.001	-0.09 (-0.57, -0.01) *p* = 0.003	0.00 (-0.09, 0.14) *p* = 0.91
Left side Maximum pupil size, mm, median (IQR)	2.44 (2.08, 3.19)	2.21 (2.02, 2.78)	2.31 (2.02, 3.19)	0.19 (0.09, 0.46) *p* < 0.001	-0.16 (-0.31, -0.04) *p* = 0.001	0.04 (-0.04, 0.17) *p* = 0.33
Right side Minimum pupil size, mm, median (IQR)	2.02 (1.68, 2.30)	2.04 (1.69, 2.32)	1.99 (1.69, 2.47)	0.01 (-0.1, 0.12) *p* = 0.78	-0.02 (-0.18, 0.12) *p* = 0.86	-0.01 (-0.12, 0.08) *p* = 0.94
Left side Minimum pupil size, mm, median (IQR)	1.81 (1.67, 2.36)	1.96 (1.68, 2.47)	1.86 (1.68, 2.40)	-0.05 (-0.11, 0.03) *p* = 0.18	0.03 (-0.07, 0.12) *p* = 0.70	-0.09 (-0.21, 0.12) *p* = 0.99
Right side Relative change, %, median (IQR)	18.0 (15.5, 26.5)	11.0 (8.5, 16.5)	17.5 (14.5, 27.0)	7.0 (2.5, 12.0) *p* < 0.001	-5.5 (-10.5, -4.0) *p* < 0.001	0.0 (-1.5, 2.5) *p* = 0.96
Left side Relative change, %, median (IQR)	19.5 (16.5, 28.5)	9.0 (7.0, 16.5)	18.5 (15.5, 25.5)	9.5 (6.0, 12.5) *p* < 0.001	-8.0 (-10.5, -6.0) *p* < 0.001	0.0 (-1.0, 5.0) *p* = 0.15
Right side Constriction velocity, mm/s, mean (SD)	1.18 (0.65)	0.79 (0.53)	1.21 (0.64)	0.39 (0.47) *p* = 0.001	-0.42 (0.45) *p* < 0.001	-0.03 (0.23) *p* = 0.62
Left side Constriction velocity, mm/s, mean (SD)	1.15 (0.63)	0.73 (0.56)	1.06 (0.64)	0.42 (0.42) *p* < 0.001	-0.33 (0.35) *p* < 0.001	0.09 (0.24) *p* = 0.05
Right side Maximum constriction velocity, mm/s, median (IQR)	1.57 (1.19, 2.38)	1.05 (0.76, 1.33)	1.51 (1.21, 2.34)	0.54 (0.29, 1.13) *p* < 0.001	-0.57 (-1.19, -0.23) *p* < 0.001	-0.02 (-0.15, 0.21) *p* = 0.54
Left side Maximum constriction velocity, mm/s, median (IQR)	1.43 (1.23, 1.97)	0.92 (0.58, 1.40)	1.34 (1.19, 2.04)	0.67 (0.31, 0.95) *p* < 0.001	-0.46 (-0.86, -0.34) *p* < 0.001	0.01 (-0.11, 0.17) *p* = 0.50
Right side Latency, s, median (IQR)	0.23 (0.20, 0.30)	0.27 (0.23, 0.32)	0.23 (0.20, 0.30)	-0.02 (-0.10, 0.00) *p* = 0.09	0.00 (0.00, 0.07) *p* = 0.11	-0.02 (-0.04, 0.04) *p* = 0.87
Left side Latency, s, median (IQR)	0.23 (0.20, 0.30)	0.27 (0.20, 0.30)	0.27 (0.20, 0.27)	0.00 (-0.05, 0.05) *p* = 0.97	0.02 (-0.07, 0.04) *p* = 0.64	-0.02 (-0.04, 0.04) *p* = 0.95
Right side Dilation velocity, mm/s, mean (SD)	0.42 (0.26)	0.36 (0.29)	0.42 (0.23)	0.06 (0.19) *p* = 0.09	-0.06 (0.19) *p* = 0.09	0.00 (0.10) *p* = 1.00
Left side Dilation velocity, mm/s, mean (SD)	0.42 (0.26)	0.31 (0.32)	0.44 (0.32)	0.11 (0.22) *p* = 0.02	-0.13 (0.14) *p* < 0.001	-0.02 (0.15) *p* = 0.52

**Table 3:** Means or medians of pupillometry values for each observation. DC1.1 first observation under first set of dark conditions, DC1.2 s observation under first set of dark conditions, BC1.1 first observation under bright conditions, BC1.2 s observation under bright conditions, DC2.1 first observation under second set of dark conditions, DC2.2 s observation under second set of dark conditions, IQR interquartile range, NPi neurological pupil index, SD standard deviation

Parameter	DC1.1	DC1.2	BC1.1	BC1.2	DC2.1	DC2.2
Right side NPi, median (IQR)	4.6 (4.5, 4.7)	4.5 (4.1, 4.7)	4.2 (3.9, 4.6)	4.3 (4.0, 4.5)	4.5 (4.4, 4.7)	4.4 (4.2, 4.7)
Left side NPi, median (IQR)	4.7 (4.4, 4.7)	4.6 (4.2, 4.7)	4.1 (3.7, 4.5)	4.2 (3.8, 4.5)	4.6 (4.4, 4.7)	4.6 (4.1, 4.6)
Right side Maximum pupil size, mm, median (IQR)	2.42 (2.23, 3.03)	2.44 (2.16, 3.01)	2.29 (2.02, 2.79)	2.19 (2.03, 2.54)	2.41 (2.17, 3.16)	2.49 (2.17, 3.08)
Left side Maximum pupil size, mm, median (IQR)	2.44 (2.08, 3.19)	2.31 (2.04, 3.20)	2.21 (2.02, 2.78)	2.19 (1.91, 2.50)	2.31 (2.02, 3.19)	2.34 (2.05, 3.50)
Right side Minimum pupil size, mm, median (IQR)	2.02 (1.68, 2.30)	2.03 (1.71, 2.57)	2.04 (1.69, 2.32)	1.92 (1.64, 2.16)	1.99 (1.69, 2.47)	2.03 (1.74, 2.48)
Left side Minimum pupil size, mm, median (IQR)	1.81 (1.67, 2.36)	1.84 (1.64, 2.38)	1.96 (1.68, 2.47)	1.88 (1.67, 2.24)	1.86 (1.68, 2.40)	1.87 (1.68, 2.72)
Right side Relative change, %, median (IQR)	18.0 (15.5, 26.5)	18.0 (14.0, 26.0)	11.0 (8.5, 16.5)	12.5 (9.5, 17.5)	17.5 (14.5, 27.0)	16.5 (10.5, 24.5)
Left side Relative change, %, median (IQR)	19.5 (16.5, 28.5)	17.0 (12.5, 27.5)	9.0 (7.0, 16.5)	11.5 (9.5, 16.0)	18.5 (15.5, 25.5)	16.5 (13.5, 26.0)
Right side Constriction velocity, mm/s, mean (SD)	1.18 (0.65)	1.15 (0.59)	0.79 (0.53)	0.67 (0.38)	1.21 (0.64)	1.19 (0.67)
Left side Constriction velocity, mm/s, mean (SD)	1.15 (0.63)	1.04 (0.60)	0.73 (0.56)	0.67 (0.43)	1.06 (0.64)	1.09 (0.73)
Right side Maximum constriction velocity, mm/s, median (IQR)	1.57 (1.19, 2.38)	1.53 (1.13, 2.45)	1.05 (0.76, 1.33)	0.87 (0.66, 1.46)	1.51 (1.21, 2.34)	1.47 (1.12, 2.31)
Left side Maximum constriction velocity, mm/s, median (IQR)	1.43 (1.23, 1.97)	1.41 (1.25, 1.98)	0.92 (0.58, 1.40)	0.94 (0.64, 1.44)	1.34 (1.19, 2.04)	1.41 (1.09, 1.89)
Right side Latency, s, median (IQR)	0.23 (0.20, 0.30)	0.27 (0.23, 0.30)	0.27 (0.23, 0.32)	0.27 (0.20, 0.30)	0.23 (0.20, 0.30)	0.23 (0.23, 0.30)
Left side Latency, s, median (IQR)	0.23 (0.20, 0.30)	0.23 (0.23, 0.27)	0.27 (0.20, 0.30)	0.27 (0.20, 0.30)	0.27 (0.20, 0.27)	0.23 (0.20, 0.27)
Right side Dilation velocity, mm/s, mean (SD)	0.42 (0.26)	0.38 (0.24)	0.36 (0.29)	0.32 (0.23)	0.42 (0.23)	0.37 (0.25)
Left side Dilation velocity, mm/s, mean (SD)	0.42 (0.26)	0.41 (0.25)	0.31 (0.32)	0.28 (0.19)	0.44 (0.32)	0.41 (0.32)

## Discussion

This study shows that NPi, alongside several of the parameters it is calculated from, are affected by ambient light levels in a mixed critical care population. This is in line with findings from the only previous study comparing NPi in dark and bright conditions using the Neuroptics^®^ NPi-200 (Neuroptics, Irvine CA, USA) pupillometer [5]. In a small study using a Neurolight pupillometer (IDMed, France), no differences in PLR could be detected between dark and bright ambient light conditions, although this pupillometer utilizes an opaque rubber cup, shutting out ambient light from the examined eye [7].

Quantitative pupillometry is an important improvement of the neurological exam, providing quantitative measurements of the PLR, rather than arbitrary statements. Quantitative pupillometry has been shown to have varying potential applications. It has been extensively researched as a tool to estimate ICP or screen for elevated ICP, with varying results and weak, albeit significant, associations between NPi and ICP [8–14]. Despite the weak associations with ICP, due to its ease of use, QP remains a promising tool in screening for elevated ICP, particularly in low resource settings [15]. It has shown some potential as an early warning sign of delayed cerebral ischemia after aneurysmal subarachnoid hemorrhage [16–18], although this has been called into question by recent results and more studies are needed [19]. The NPi is a promising tool for prognostication of neurological outcome after cardiac arrest, with lower values consistently being associated with poor outcomes [20–25]. Although optimal cut-offs to predict poor outcome vary between studies, QP is recommended as part of the multimodal neurological prognostication post cardiac arrest in recent guidelines [26]. Values from QP has further been suggested to be able to predict delirium, and has shown some promise in this regard in a few studies, albeit with weak associations between QP values and later onset of delirium [27–30].

With their nuclei in the midbrain [1], function of the optic and oculomotor nerve, as assessed by quantitative pupillometry, is an important tool to monitor patients with infratentorial and/or brainstem lesions. Also, QP has shown potential in assessing analgesia in patients unable to score their pain on visual or numeric scales, as well as in predicting the pain response to different stimuli [31–38]. Still, unnecessary high doses of perioperative opioids have also been shown when using QP guided titration of analgesia [39]. Finally, absence of PLR is one of the neurological criteria of brain death [40]. With such varying applications, QP is widely used in large patient groups and may in some circumstances influence high stakes decisions. The issue of correct usage and interpretation is therefore highly relevant for both clinical practice and future studies. With significant but weak associations for many of the applications mentioned above, there is reason to clean up noisy signals as far as possible in future studies. Ambient light levels are seldom reported in studies of these QP applications and may be one source of noise that could be reduced.

The median difference in NPi between bright and dark conditions can be considered small in our study. Previous results indicate a standard deviation of up to 0.8 for NPi in a large multicenter study with repeated observations in neurocritical care patients [41]. Still, that study was performed on register data based on data from routine QP exams. Although the authors stratified by GCS intervals to adjust for severity of neurological status, they comment that GCS is a poor indicator of neurological disease or injury severity in a largely sedated cohort. Likewise, that study did not adjust for diagnosis, drugs or dosages of sedation, infusions with catecholamines, or levels of ICP. All these factors may affect the NPi. Since drugs, dosages and ICP all are dynamic variables, they are likely to increase within-patient variability of NPi. Further, standardization of ambient light was not specified in the protocol for the register used in that study [42]. Ergo, the effect of ambient light reported in our study may in fact contribute to the wide standard deviations shown in that study. Also, it should be noted that in 25% of the patients in our study, the NPi decreased by 0.6 or more on the right side and 0.7 or more on the left side, in bright conditions. Increases or decreases of that magnitude could well be misinterpreted as a deterioration or improvement of neurological status, particularly in patients hovering around the 3.0 cut-off for normal NPi. Likewise, even small differences in NPi may prove crucial in clinical diagnosis of brain death, when the PLR is considered as either absent, with an NPi of 0.0, or present, with any NPi > 0.0. Although there were intra-individual fluctuations in NPi at the same levels of ambient light that in some cases may be considered clinically significant, these fluctuations were overall small and significantly smaller than the differences between lighting conditions. Hence, the differences in NPi between lighting conditions did not arise from within-individual fluctuations.

Our study has several major limitations. Although it is the largest study yet evaluating the effect of ambient light on NPi, it remains a very small, single center study with merely 20 participants. We also used a convenience sample. Thus, there are important limitations to the generalizability of our findings. Since not all patients included were monitored with invasive ICP measurement, we cannot rule out a potential effect of ICP fluctuations on NPi. Still, previous findings of the weak association between ICP and NPi indicate that the effects of ICP fluctuations would likely be negligible, unless the ICP fluctuations were to be quite large [14, 43]. Further, such fluctuations are improbable to introduce confounding since they would likely not be associated with the differences in ambient light. Other potential stimuli that may affect the PLR are painful stimuli and fever. We did not gather any data on these. Although they need to be considered as limitations to the interpretation of our results, we believe it unlikely that pain or fever should have changed significantly during the few minutes it took to perform these measurements in any given patient. In the unlikely event of large changes in pain or body temperature, we find it even less likely that they should be associated with the level of ambient light and consider it improbable that these factors should have confounded our results.

Another important limitation with this study is the short interval between observations. Retinal adaptation to changes in light conditions may occur, and have measurable effects on the PLR, up to 20 min after the conditions have changed [44]. For obvious ethical reasons, we needed to avoid interference with ongoing patient care and treatment. The conditions of intensive care patients are dynamic and interventions to stabilize patients are frequent, such as bolus doses of sedatives and opioids, or changes in doses of norepinephrine. Since all of these may hypothetically affect QP values, we needed to avoid that such necessary pharmacological interventions, or other stimuli, occurred between observations. For these two reasons, we opted for short observation intervals of only two minutes between observations. These short wash out periods between observations may in part explain the relatively small differences we found, compared to Ong et al. who used longer wash out periods [5]. Thus, the differences between bright and dark conditions may be underestimated in our study. Also, we did not perform measurements of ambient light simultaneous to our pupillometry measurements. Although our post-hoc measurement of ambient light levels was performed in conditions mimicking those of the study setting, the light levels reported in the methods section may differ from those that occurred during gathering of data. We also would like to comment that the dark conditions in our study likely are darker than most settings in which pupillometry is performed in clinical practice. The bright conditions, with a post-hoc measurement at 301 lx, represent common indoor lighting conditions and are likely to occur quite often during pupillometry in clinical practice. Finally, we urge caution in interpretation of the secondary analyses presented here. This dataset is small and the distributions are wide for some parameters. This study was not designed to exhaustively analyze the effect of ambient light levels on all pupillometry values. The secondary analyses presented may be used to generate hypotheses but should not guide clinical practice.

One of the strengths with this study is that it was performed in a mixed critical care cohort and not in healthy volunteers. Previous results indicate that NPi does not differ significantly between ambient light conditions in healthy volunteers [5]. The clinical utility of pupillometry is largely in emergency and critical care settings. Hence our cohort is more representative of the population of interest than healthy volunteers would have been. Although the heterogeneity of the cohort with regards to diagnoses could be considered a drawback, we rather see this as a strength with the study. It does not limit the results to one narrow diagnosis group but indicates that an effect of ambient light on QP values occurs across varying groups of intensive care patients. The crossover design chosen was therefore well suited for our research question. With patients being their own identical controls, this adjusts for the noisy signal we would see in a similar comparison on a cohort level.

The clinical and scientific implication of this study is that QP should be performed with standardized levels of ambient light when trends are to be assessed. Standardized levels of ambient light may also increase accuracy in detecting neurological deterioration. This is particularly important in patients lacking multimodal and/or invasive neuromonitoring, where pupillometry can be the only neuromonitoring device available. Likewise, it is important in patients with infratentorial and/or brainstem lesions, were supratentorial invasive monitoring may be of little value [45].

Alternatively, pupillometers with opaque cups, shielding the examined eye from ambient light, may be a solution. Still, the difference between QP values under bright and dark conditions could potentially be of diagnostic value per se, similar to how blood pressure measurements both in supine and standing positions are of value. However, no such studies have been performed on QP. It may be that QP under both bright and dark conditions provide more information than either one of them does alone.

Smartphone applications for QP are available and have shown potential value in emergency settings [46]. This development makes pupillometry widely available in low resource settings. Further, it has been shown to enable automatic correction of PLR measurements for ambient light with promising results [47]. Still, the agreement between smartphone application QP and well-studied and standardized measurements, such as the NPi, seems poor [48]. Although a promising development, these techniques may still be considered immature. Nonetheless, since ambient lighting often cannot be controlled in emergency or prehospital settings, our findings suggest that correction for ambient light could be a welcome addition to the QP technique, enabling PLR exams to be of greater clinical use in broader contexts.

## Conclusion

In conclusion, our study suggests that most parameters of quantitative pupillometry, including NPi, are affected by ambient light in critical care patients. This may have clinical as well as scientific implications. When comparing QP values, or looking at trends of QP values, we suggest standardized ambient light settings. Still, comparisons of QP values from both bright and dark conditions may also be of value in some settings. In settings where standardized ambient light is not achievable, automatic correction for ambient light in smartphone applications for PLR measurement may be a promising development of automatic pupillometry.

## Data Availability

No datasets were generated or analysed during the current study.
